# Life-Course Circumstances and Frailty in Old Age Within Different European Welfare Regimes: A Longitudinal Study With SHARE

**DOI:** 10.1093/geronb/gbz140

**Published:** 2019-10-30

**Authors:** Bernadette Wilhelmina Antonia Van Der Linden, Stefan Sieber, Boris Cheval, Dan Orsholits, Idris Guessous, Rainer Gabriel, Martina Von Arx, Michelle Kelly-Irving, Marja Aartsen, David Blane, Matthieu P Boisgontier, Delphine Courvoisier, Michel Oris, Matthias Kliegel, Stéphane Cullati

**Affiliations:** 1 Swiss NCCR “LIVES - Overcoming Vulnerability: Life Course Perspectives”; 2 Center for the Interdisciplinary Study of Gerontology and Vulnerability; 3 Department of Readaptation and Geriatrics, University of Geneva, Switzerland; 4 Department of Community Medicine, Primary Care and Emergency Medicine, Geneva University Hospitals, Switzerland; 5 Department of Ambulatory Care and Community Medicine, University of Lausanne, Switzerland; 6 ZHAW, Zurich University of Applied Sciences, Switzerland; 7 INSERM, UMR1027, Toulouse, France; 8 Université Toulouse III Paul-Sabatier, UMR1027, Toulouse, France; 9 NOVA - Norwegian Social Research, Center for Welfare and Labor Research, Oslo, Norway; 10 International Centre for Life Course Studies in Society and Health, Department of Epidemiology and Public Health, University College London, UK; 11 Institute of Sociological Research, University of Geneva, Switzerland

**Keywords:** Childhood disadvantage, Health outcomes, Socioeconomic status, Successful aging

## Abstract

**Objectives:**

This study aimed to assess whether cumulative disadvantage in childhood misfortune and adult-life socioeconomic conditions influence the risk of frailty in old age and whether welfare regimes influence these associations.

**Method:**

Data from 23,358 participants aged 50 years and older included in the longitudinal SHARE survey were used. Frailty was operationalized according to Fried’s phenotype as presenting either weakness, shrinking, exhaustion, slowness, or low activity. Confounder-adjusted mixed-effects logistic regression models were used to analyze associations of childhood misfortune and life-course socioeconomic conditions with frailty.

**Results:**

Childhood misfortune and poor adult-life socioeconomic conditions increased the odds of (pre-)frailty at older age. With aging, differences narrowed between categories of adverse childhood experiences (driven by Scandinavian welfare regime) and adverse childhood health experiences (driven by Eastern European welfare regime), but increased between categories of occupational position (driven by Bismarckian welfare regime).

**Discussion:**

These findings suggest that childhood misfortune is linked to frailty in old age. Such a disadvantaged start in life does not seem to be compensated by a person’s life-course socioeconomic trajectory, though certain types of welfare regimes affected this relationship. Apart from main occupational position, our findings do not support the cumulative dis/advantage theory, but rather show narrowing differences.

One of the major social challenges related to the increase in life expectancy is the rise of chronic conditions and multi-morbidity in older age. Frailty, a syndrome of increased vulnerability to stressors caused by cumulative decline across biological systems, is thus a relevant public health issue for societies worldwide (Clegg, Young, Iliffe, Rikkert, & Rockwood, 2013; Fried et al., 2001). An issue is the often perceived social patterning of frailty across time and societies (Mackenbach, 2012; Marmot, 2003), indicating unequal risk of becoming frail. Moreover, these inequalities tend to widen in older age (Etman, Kamphuis, van der Cammen, Burdorf, & van Lenthe, 2015; Franse et al., 2017; van der Linden et al., 2019). The reason for this widening is not known, but from a life-course perspective, the relationship between childhood conditions and later life health may be explained by the cumulative dis/advantage (CDA) theory, defined as the ‘systemic tendency for interindividual divergence in a given characteristic (e.g., money, health, or status) with the passage of time’ (Dannefer, 2003). This theory posits a widening social gradient of frailty in old age.

Based on the CDA hypothesis, three aspects of the theory can be tested in the context of frailty. First, is there a social gradient of frailty in old age and are there growing differences in frailty trajectories with aging? Second, we will investigate the principle of life-course reflexivity (Dannefer, 2018) by focusing not only on childhood effects, but also targeting interactive dynamics that occur between an individual and his or her social system across the life-course by including socioeconomic conditions in adulthood, thereby examining changes in mid- and later life. Third, as welfare policies regulate the level to which individual life-courses are affected by macro-level changes (Dannefer, 2018; Leisering, 2003; Sieber et al., 2019), we test the CDA mechanism at the micro (childhood misfortune and adult-life socioeconomic conditions (SEC)) as well as the macro (welfare regimes) level.

Childhood has been shown to influence later life health through biological and psychosocial pathways and represents a period in which people are most vulnerable to external influences (Blane et al., 2013; Kuh et al., 2013; Stringhini et al., 2015). Regarding frailty, several studies suggested that its key risk is rooted in childhood socioeconomic conditions (CSC) (Alvarado, Zunzunegui, Beland, & Bamvita, 2008; Gale, Booth, Starr, & Deary, 2016; Hughes et al., 2017; van der Linden et al., 2019). Other childhood circumstances often linked to negative health outcomes later in life are traumatic events referred to as adverse childhood experiences (ACE) (Nurius, Fleming, & Brindle, 2019; Wade et al., 2016). However, studies examining the effect of ACE on the risk of frailty in older age are lacking. Moreover, later life health is not only influenced by childhood health, but also by poor socioeconomic and adverse psychosocial conditions during early life, as already reported for outcomes related to frailty such as successful aging and functional health (Brandt, Deindl, & Hank, 2012; Haas, 2008; Huang, Soldo, & Elo, 2011). Based on this, we expect that childhood exposure to adverse conditions increases the risk of frailty.

In addition, growing up in adverse circumstances can influence later life health through a linkage with structural factors in several domains that are important in the studied cohort. During the period the studied cohort was growing up, disadvantaged socioeconomic conditions often went hand in hand with living in poor environmental and low-income conditions, restricted access to high-quality education, health care, and social network (Doku, Acacio-Claro, Koivusilta, & Rimpela, 2018; Sharpe et al., 2018). Several socioeconomic factors in adulthood such as education, occupational class and wealth were found to contribute to persisting health and frailty inequalities in old age (Santos-Eggimann, Cuenoud, Spagnoli, & Junod, 2009; Stolz, Mayerl, Waxenegger, Rasky, & Freidl, 2017; van der Linden et al., 2019). Therefore, we expect to find that lower education, occupation, and income will be associated with a greater risk of frailty in later life.

When taking a life-course perspective, not only individual factors, but also macro level influences, such as welfare regimes should be considered, as nation states’ policies may influence accumulation of dis/advantage through pension systems and social benefits (Oris, Gabriel, Ritschard, & Kliegel, 2017). More supportive welfare regimes are more likely to favor redistribution and absorb the impact of material shortfalls through the provision of higher benefits (Bartley, Blane, & Montgomery, 1997; Raphael & Bryant, 2015). In contrast, less generous welfare regimes may increase the impact of accumulated disadvantages. Ferrera’s typology, augmented by the Eastern European welfare regime, can be used as a basis for grouping countries into welfare regimes based on how social benefits are granted and organized, with different roles of the state, family, and market in the provision of welfare (Eikemo, Bambra, Judge, & Ringdal, 2008; Ferrera, 1996; Sieber et al., 2019). The Bismarckian welfare regime is known for its ‘status differentiating’ welfare programs where benefits are related to earnings and administered by employers. This regime is minimally redistributive (Eikemo et al., 2008). Conversely, the Scandinavian welfare regime aims at promoting social equality through a redistributive social security system (Eikemo et al., 2008). The Southern European welfare regime is more fragmented in terms of welfare provision and strongly relies on the family (Eikemo et al., 2008; Ferrera, 1996). The Eastern European welfare regime is less easy to capture in a life-course perspective since it can currently be characterized by limited health service provision and poor population health, but when the elderly we study were children, they grew up in a care and school system inspired by an egalitarian ethos (Eikemo et al., 2008). Considering these characteristics, we hypothesize that the Bismarckian welfare regime will be the least able to compensate accumulation of dis/advantage, whereas the Scandinavian welfare regime will be more efficient. For Southern and East European regimes, we expect less clear results.

In line with the three aspects of the CDA theory, this study has three objectives. First, to examine the associations of different forms of childhood misfortune (poor socioeconomic conditions, adverse experiences, and poor health) with levels and trajectories of frailty over aging. Second, to examine the role of adult-life SEC (education, main occupation, and satisfaction with household income) in the association of childhood adversities with levels and trajectories of frailty at older age. Third, to assess the role of welfare regimes in these associations.

## Method

### Study Design and Population

Data from the longitudinal Survey of Health, Ageing, and Retirement in Europe (SHARE) were used. SHARE was designed to investigate population aging processes (Borsch-Supan et al., 2013). We included six waves of data that were collected every 2 years between 2004 and 2016. Participants were eligible for the analyses if they participated in the third wave and had at least one complete measure of frailty in wave 1, 2, 4, 5 or 6. Participation in the third wave was necessary since retrospective life-course data on socioeconomic conditions was only collected in wave 3 (SHARELIFE).

### Frailty

To construct the frailty variable, the attributes from the phenotype of frailty were used; shrinking, weakness, exhaustion, slowness, and low activity (Fried et al., 2001). The operationalization was adapted to the provided information in SHARE, for which we adhered to Santos-Eggimann et al.’s (2009) proposition of the operationalization of frailty. It was constructed by selecting the most suitable metric and has been tested and validated in SHARE (van der Linden et al., 2019; Macklai, Spagnoli, Junod, & Santos-Eggimann, 2013; Romero-Ortuno, 2013). For shrinking, the question, “What has your appetite been like” was used and the criterion was fulfilled when participants reported a “diminution in desire for food” or, in the case of an unclear response to this question, the answer “less” to the follow-up item “So have you been eating more or less than usual?”. Weakness was operationalized using grip strength measures and the highest out of four dynamometer measures was analyzed. Cutoffs were calculated for each wave separately, stratified by gender and body mass index quartiles (Fried et al., 2001) and the criterion was fulfilled by the weakest 20% in each category. The question, “In the last month, have you had too little energy to do things you wanted to do?” was used to define exhaustion. The slowness attribute was operationalized using mobility questions, as SHARE measured walking speed only for individuals aged 75 or older. Previous analyses showed that low speed and positive answers to either of the following two items were strongly associated: “Because of a health problem, do you have any difficulty [expected to last three or more months] walking 100 meters” or “…climbing one flight of stairs without resting” (Santos-Eggimann et al., 2009). The question “How often do you engage in activities that require a low or moderate level of energy such as gardening, cleaning the car, or going for a walk?” was used for the low activity attribute which was fulfilled for individuals answering either “one to three times a month” or “hardly ever or never”.

A score ranging from zero to five was created, based on fulfillment of the attributes. Individuals with zero points were classified as non-frail, one or two points as pre-frail, and three or more points as frail (Fried et al., 2001). To create a binary outcome, pre-frail and frail states were combined to (pre-)frail as opposed to non-frail (van der Linden et al., 2019).

### Childhood Misfortune

#### Adverse childhood experiences

ACE were defined as a set of traumatic events (emotional, physical, or linked to household dysfunction) that occurred during childhood (from 0 to 15 years) and that were outside a child’s control (Felitti et al., 1998). The following indicators for specific ACE matching this definition were selected; child in care (living in a children’s home or with a foster family), parental death (father, mother, or both), parental mental illness, parental drinking abuse, period of hunger, and property taken away. An ACE score ranging from 0 to 7 was created by combining the six indicators: one point for presence of each indicator and two points when a participant lost both parents in childhood. To examine the overall chronic stress response induced by having experienced any ACE, we dichotomized the score into participants who experienced no ACE (i.e., participants who only answered “no”) versus participants who experienced at least one ACE (i.e., participants who answered “yes” at least once) (Barboza Solís et al., 2015; Cheval et al., 2019). When information was missing, the score was computed using the non-missing data of all available items.

#### Adverse childhood health experiences

The following indicators of childhood health problems up until the age of 15 were included; long hospitalization (hospitalization for at least one month), multiple hospitalizations (more than three times within a 12-month period), childhood illness (including polio, asthma, or meningitis/encephalitis), serious health conditions (including severe headaches, psychiatric problem, fractures, heart trouble, cancers), and physical injury that has led to permanent handicap, disability or limitation in daily life (Cheval et al., 2019). The adverse childhood health experiences (ACHE) variable was created by computing a binary variable of participants who experienced no ACHE versus participants who experienced at least one AHCE. For missing information, the score was computed using the non-missing data of the available items.

#### Childhood socioeconomic conditions

Four binary indicators of socioeconomic conditions at age 10, according to Wahrendorf and Blane’s (2015) measure of childhood circumstances were used; occupational position of the main breadwinner, number of books at home, overcrowding, and housing quality. The five-level categorical CSC variable was constructed by combining these indicators creating a score from “most disadvantaged” to “most advantaged”. The same score was used in a previous article which provides more detailed information about the coding of this variable (Cheval et al., 2018).

### Adult-Life Socioeconomic Conditions

As indicators of adult-life socioeconomic conditions, variables were included that represent young adult life, middle age, and old age, respectively; highest educational attainment (primary, secondary, or tertiary) during follow-up, main occupational position based on the skill classification of the main job over the life-course (high skill vs low skill), and satisfaction with current household income, using the question “Is the household able to make ends meet?” (ranging from 1 “with great difficulty” to 4 “easily”). To keep as many observations as possible, the mode over follow-up for each individual was computed.

### Welfare Regimes

Countries were classified into four welfare regimes based on the classification proposed by Eikemo and colleagues (2008); Scandinavian (Denmark and Sweden), Bismarckian (Austria, Belgium, France, Germany, the Netherlands, Switzerland), Southern European (Greece, Italy, Spain), and Eastern European (Czech Republic and Poland). Welfare regime was measured at follow-up as a proxy of individuals’ life-course regime.

### Potential Confounders and Predictors

All analyses were adjusted for age, sex, attrition (no dropout, dropout, deceased), and birth cohort (1919–1928, 1929–1938 [Great Depression], 1939–1945 [Second World War], and post-1945) as these variables have been shown to be related to later life health (Cheval et al., 2019; van der Linden et al., 2019; Sieber et al., 2019). Final models were additionally adjusted for other possible health- and lifestyle related predictors of frailty as final adjustment; living with partner (yes, no), delayed recall memory and verbal fluency as indicators of cognitive functioning, smoking (ever, not), number of chronic conditions (including stroke, heart attack, hypertension, diabetes, cancer, Parkinson’s disease, and asthma), difficulties with ADL (score range zero to five), and difficulties with instrumental activities of daily living (IADL, score range zero to five) (Gale et al., 2016; Hughes et al., 2017; van der Linden et al., 2019).

### Statistical Analyses

We used logistic mixed-effects models to analyze the data (Boisgontier & Cheval, 2016). The models’ Bayesian Information Criterion as well as likelihood tests revealed that the best random structure was random intercepts. Age was centered at the beginning of the trajectory (i.e., 50 years) and divided by 10 so that the coefficient yielded effects of increase in the odds of being frail over a 10-year period. For the time varying covariates, we used the mode in order to reduce the loss of observations. Model 1a tested the association between childhood misfortune (CSC, ACE, ACHE) and the odds of being frail. In addition, model 1a included interaction terms between age and childhood misfortune to test whether the differences between childhood categories were growing or narrowing as people age. In model 2a we added the adult-life SEC (education, main occupational position, satisfaction with household income) and their respective interactions. Coherently with the latest formulation of the CDA theory that posits the capacity of the welfare regime to moderate associations of early- and adult-life experiences with old age outcomes (Dannefer, 2018), we tested triple interactions between age, predictors and welfare regime to assess whether this was also the case for frailty. Since the interactions were significant, we stratified the models by welfare regime, that is, models 1b and 2b which correspond to the unstratified models 1a and 2a (Supplementary Table S2 for Scandinavian and Bismarckian welfare regimes and S3 for Southern and Eastern European welfare regimes, see Supplementary Material). Models 1a, 1b, 2a, and 2b were adjusted for sex, birth cohort, and sample-attrition. Models 3a and 3b additionally includes health- and lifestyle variables (without interactions) to fully adjust the models (Supplementary Table S4, see Supplementary Material).

Finally, we performed a series of sensitivity analyses by excluding participants older than 90 years (a), who died during the survey (b), and those who dropped out (c), and finally testing the effect of each ACE on the risk of frailty in later life (d). We also ran stratified analyses by sex, since the prevalence of frailty is expected to differ between men and women. Statistical analyses were performed using R with the lme4 and lmerTest packages (Bates, Mächler, Bolker, & Walker, 2015; Kuznetsova, 2016; R Core Team, 2017).

## Results

### Participant Characteristics

**Figure 1.**
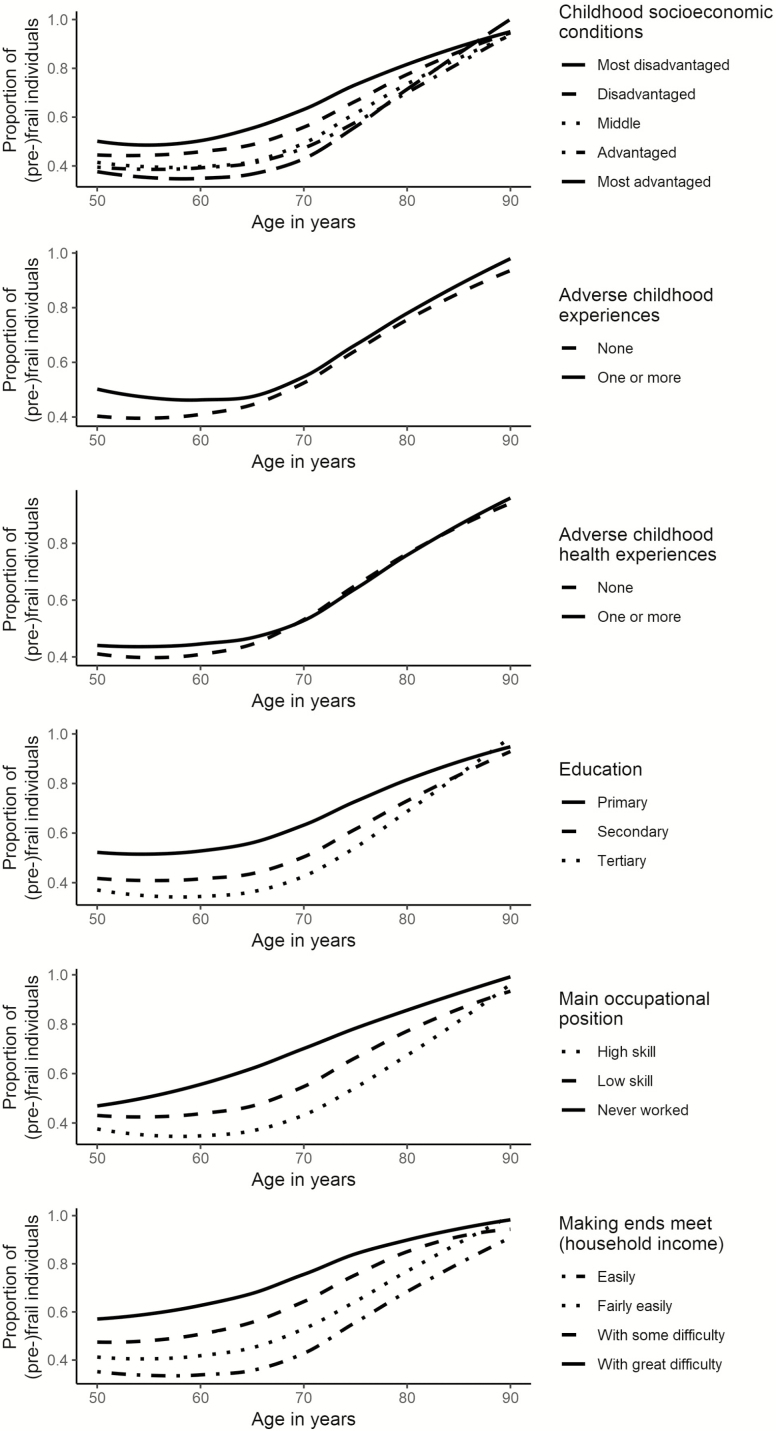
Descriptive plot of observed evolution over aging of (pre-)frailty proportions by childhood misfortune and adult-life socioeconomic conditions

The final sample consisted of 23 358 participants (56% female) with a mean age of 60.6 years in the nonfrail and 64.6 in the (pre-)frail group (see Table 1 for the total sample and Supplementary Table S1 for the sample stratified by welfare regime). Most participants did not experience any ACE (79%) and ACHE (75%). A gradient in CSC and ACE was observed: the percentage of (pre-)frail people was 41.4% in the most advantaged category compared to 62.8% in the most disadvantaged and 48.5% in the no ACE category compared to 54.5% in the category with at least one ACE. A similar gradient was found for the adult-life indicators: the lower the educational attainment, occupational position, and satisfaction with household income, the higher the proportion of (pre-)frail. Descriptive trajectories of frailty for each childhood misfortune and adult-life SEC show an overall parallel evolution until 70 years (Figure 1). After the age of 70, differences between the categories narrow, mainly for the childhood misfortune variables.

**Table 1.** Participant Characteristics

	Nonfrail	(Pre-)frail
	*N* (%)	*N* (%)
Age, mean (SD)	60.6 (7.8)	64.6 (9.8)
Sex		
Female	5,954 (45.8)	7,038 (54.2)
Male	5,777 (55.7)	4,589 (44.3)
Welfare regime		
Scandinavian	2,019 (56.2)	1,571 (43.8)
Bismarckian	5,559 (55.3)	4,495 (44.7)
Southern European	2,782 (42.3)	3,795 (57.7)
Eastern European	1,371 (43.7)	1,766 (56.3)
Adverse childhood experiences		
None	9,465 (51.5)	8,914 (48.5)
At least one	2,266 (45.5)	2,713 (54.5)
Adverse childhood health experiences		
None	8,723 (50.1)	8,698 (49.9)
At least one	3,008 (50.7)	2,929 (49.3)
Childhood socioeconomic conditions		
Most disadvantaged	1,598 (37.2)	2,697 (62.8)
Disadvantaged	2,810 (48.0)	3,041 (52.0)
Middle	4,097 (54.3)	3,454 (45.7)
Advantaged	2,448 (56.5)	1,886 (43.5)
Most advantaged	778 (58.6)	549 (41.4)
Education		
Primary	2,560 (36.9)	4,380 (63.1)
Secondary	6,396 (53.9)	5,462 (46.1)
Tertiary	2,775 (60.9)	1,785 (39.1)
Main occupational position		
High skill	3,207 (60.0)	2,138 (40.0)
Low skill	7,876 (48.9)	8,242 (51.1)
Never worked	648 (34.2)	1,247 (65.8)
Satisfaction with household income		
Easily	5,223 (59.4)	3,567 (40.6)
Fairly easily	3,650 (51.1)	3,492 (48.9)
With some difficulty	2,093 (41.7)	2,924 (58.3)
With great difficulty	765 (31.8)	1,644 (68.2)

*Note:* SD = standard deviation. *N* nonfrail = 11,731, *N* (pre-)frail = 11,627.

### Effects of Childhood Misfortune on Risk of Frailty Over Aging

The results for the association of childhood misfortune with the odds of being (pre-)frail are shown for model 1a in Table 2. At the beginning of the trajectory (i.e. age 50), participants growing up in more disadvantaged CSC, having at least one ACE, or having at least one ACHE had higher odds of being (pre-)frail compared to those in more advantaged CSC and having no ACE or ACHE. The linear trajectories of higher odds of frailty over aging differed by ACHE and CSC categories. Those reporting at least one ACHE as well as respondents growing up in middle or advantaged CSC had a less steep linear increase in the odds of being (pre-)frail than those who had no ACHE and those who grew up in most disadvantaged CSC. For ACE, no differing trajectories were found.

**Table 2.** Associations of Childhood Misfortune and Adult-Life Socioeconomic Circumstances With Level and Trajectories of Frailty at Old Age

	M1a	M2a
	OR (95% CI)	OR (95% CI)
Age (10 years period)	2.47 (2.27–2.69)***	2.32 (2.05–2.63)***
At least one ACE^a^	1.31 (1.14–1.49)***	1.30 (1.14–1.48)***
At least one ACHE^b^	1.37 (1.22–1.54)***	1.40 (1.24–1.57)***
CSC^c^ (ref. Most disadvantaged)		
Disadvantaged	0.80 (0.66–0.96)*	1.00 (0.83–1.20)
Middle	0.63 (0.53–0.75)***	1.00 (0.84–1.20)
Advantaged	0.63 (0.52–0.76)***	1.17 (0.96–1.44)
Most advantaged	0.43 (0.33–0.55)***	0.92 (0.70–1.21)
Education^d^		
Secondary		1.15 (1.00–1.33)
Primary		1.35 (1.11–1.63)**
Main Occupational Position^e^		
Low skill		1.11 (0.96–1.27)
Never worked		1.04 (0.80–1.33)
Satisfaction with household income^f^		
Fairly easily		1.36 (1.20–1.55)***
With some difficulty		2.21 (1.91–2.56)***
With great difficulty		4.34 (3.55–5.31)***
**Interactions**		
Age × at least one ACE^a^	0.97 (0.91–1.05)	0.97 (0.90–1.04)
Age × at least one ACHE^b^	0.92 (0.86–0.98)*	0.92 (0.86–0.98)*
Age × CSC^c^		
Age × Disadvantaged	0.92 (0.84–1.00)	0.92 (0.84–1.01)
Age × Middle	0.87 (0.80–0.95)**	0.88 (0.80–0.96)**
Age × Advantaged	0.82 (0.74–0.90)***	0.83 (0.74–0.92)***
Age × Most advantaged	0.92 (0.81–1.06)	0.93 (0.81–1.08)
Age × Education^d^		
Age × Secondary		0.99 (0.91–1.08)
Age × Primary		1.04 (0.94–1.16)
Age × Main occupational position^e^		
Age × Low skill		1.03 (0.95–1.11)
Age × Never worked		1.20 (1.05–1.36)**
Age × Satisfaction with household income^f^		
Age × Fairly easily		1.06 (0.99–1.13)
Age × With some difficulty		1.05 (0.97–1.14)
Age × With great difficulty		0.97 (0.86–1.09)

*Note:* ACE = adverse childhood experiences; ACHE = adverse childhood health experiences; CI = confidence interval; CSC = childhood socioeconomic conditions; OR = odds ratio. All models are adjusted for sex, birth cohort and attrition. Age was centered at 50 years and divided by 10 so that the coefficients yielded the effects for a 10 year period.

^a^Adverse childhood experiences, reference category none.

^b^Adverse childhood health experiences, reference category none.

^c^Childhood socioeconomic conditions, reference category most disadvantaged.

^d^Education, reference category tertiary.

^e^Main occupational position, reference category high skill.

^f^Satisfaction with household income, reference category easily.

*p < .05. **p < .01. ***p < .001.

### Accumulation of Disadvantage Over the Life-Course

When adult-life SEC were added to the model, CSC were no longer associated with frailty, suggesting that they mediate the associations of CSC with odds of being (pre-)frail (Table 2, model 2a). Lower educational attainment and having difficulty making ends meet were associated with higher odds of being (pre-)frail. Occupational position did not have an effect on the odds of being (pre-)frail. Over aging, the trajectories for the categories of educational attainment and satisfaction with household income did not differ. After adjusting for health- and lifestyle variables, the results did not change (Supplementary Table S4, model 3a).

### Effect of Welfare Regimes on the Association of Childhood Misfortune With Frailty

When stratifying by welfare regime, differences in the associations of childhood misfortune with frailty were found (Supplementary Tables S2 and S3, model 1b). With the exception of those growing up in middle or advantaged CSC in Eastern Europe having a lower odds of being (pre-)frail compared to those in most disadvantaged CSC, associations of CSC with frailty in the other welfare regimes were no longer observed. The association between having at least one ACE and higher odds of being (pre-)frail was still significant in the Scandinavian and Bismarckian welfare regime, but not in the Southern and Eastern European welfare regimes. In the Bismarckian and Eastern European welfare regime the association of ACHE with frailty was still present, but not in the Scandinavian and Southern European welfare regimes.

The associations of the childhood misfortune indicators with linear trajectories of frailty over aging differed between welfare regimes. The trajectories in the odds of being (pre-)frail over aging by ACE changed across welfare regimes, where only respondents in the Scandinavian welfare regime having at least one ACE had a less steep increase of odds of being (pre-)frail compared to those who did not experience ACE. The association of ACHE with linear trajectories of frailty over aging (less steep decline) observed in the overall sample (model 1a) disappeared in the Scandinavian and Southern European welfare regime, but remained in the Eastern European and Bismarckian regimes. Finally, also for CSC, trajectories of frailty over aging differed by welfare regimes. In the Eastern European welfare regime respondents growing up in middle or advantaged CSC still had a less steep linear increase of odds of being (pre-)frail than those growing up in most disadvantaged CSC. This was also present in the Bismarckian welfare regime for those in the advantaged CSC, but not in the middle CSC, but was not found in the other welfare regimes.

### Effect of Welfare Regimes on the Accumulation of Disadvantage in Adult-Life

Associations of those growing up in middle or advantaged CSC in Eastern Europe having a lower odds of being (pre-)frail compared to those in most disadvantaged CSC, disappeared when adult-life SEC were taken into account. In contrast, they appeared in the Bismarckian welfare regime where the advantaged CSC showed higher odds of being (pre-)frail compared to the most disadvantaged. Lower educational attainment was associated with higher odds of being (pre-)frail in the Bismarckian welfare regime. In the other welfare regimes, no associations were found. The association of occupational position with frailty differed by welfare regime: with the exception of those being low skilled in the Scandinavian having higher odds of being (pre-)frail compared to the high skilled, no association was found. Having difficulty making ends meet with household income differed by welfare regime and was associated with higher odds of being (pre-)frail, with the exception of those in the fairly easily category in Southern and Eastern European welfare regimes.

Over aging, the trajectories of frailty in relation to educational attainment and satisfaction with household income did not differ by welfare regime. However, those having great difficulty making ends meet with household income in the Bismarckian welfare regime had a less steep increase of being (pre-)frail over aging compared to those who could easily make ends meet. For main occupational position the previously observed growing difference between high skilled and those who never worked was no longer present across the welfare regimes. However, in the Bismarckian welfare regime a growing difference between those who were low skilled and those who were high skilled was found. After adjusting for health- and lifestyle variables, the results did not markedly change (Supplementary Table S4, model 3b).

### Sensitivity Analyses

The results of the sensitivity analyses were consistent with the findings in the main analyses. The stratified analyses revealed some sex differences as well as differences with the main analyses. For men overall, having at least one ACE was not associated with odds of being (pre-)frail, whereas for women it was. For the full sample, educational attainment was not associated with odds of being (pre-)frail in women, but men having tertiary education compared to primary education had lower odds of being (pre-)frail. However, for women in the Bismarckian welfare regime, those having primary education had higher odds of being (pre-)frail compared to tertiary education. Results for the analysis testing the effect of each of the ACE measures on the risk of frailty in later life showed an effect of child in care, parental mental illness, and parental drinking abuse with higher risk of frailty in later life.

## Discussion

The aims of this study were (a) to examine the associations of different forms of childhood misfortune with frailty over aging, (b) to examine the role of adult-life SEC in the association of childhood adversities with frailty at older age, and (c) to assess the role of welfare regimes on these associations. Several novel and conceptually relevant findings were revealed.

For the first aim, we observed associations between childhood misfortune and frailty at older age: the higher the disadvantage and having had adverse experiences, the higher the odds of being (pre-)frail at the age of 50. However, differences in frailty became smaller over time for the various ACHE and CSC categories, suggesting a diminishing validity of the CDA theory with increasing age. This convergence of frailty trajectories in later life can be explained by mortality selection in old age—also referred to as the ‘age-as-leveller’ effect—or by reversible life-course processes where disadvantaged origins can be overcome by, for example, unexpected shifts in life conditions (O’Rand, 2009). When looking at ACE in relation to concepts close to frailty, previous research support our findings that people who experienced at least one ACE had higher functional limitations in adulthood and old age (Amemiya, Fujiwara, Murayama, Tani, & Kondo, 2018; Laditka & Laditka, 2018). Other research supports the idea that childhood health is associated with levels of functional limitations in adulthood (Haas, 2008; Huang et al., 2011). Our study examined these findings for the concept of frailty and extended it by looking at old age.

For the second research question on the role of adult-life SEC, results showed that lower educational attainment and having difficulty makings ends meet with household income was associated with higher odds of being (pre-)frail at the age of 50. Additionally, when taking adult-life SEC into account, the association between CSC and frailty no longer persists. This suggests that adult-life SEC captures the cumulative effect produced by CSC in relation to frailty. With respect to changes in frailty with aging, no differences were found for education and satisfaction with household income. Our findings corroborate previous cross-sectional studies on SEC and frailty showing that poorer CSC and adult-life SEC are associated with higher risk of frailty in older age (Alvarado et al., 2008; Dury et al., 2017; Gale et al., 2016; Landos et al., 2019). Our study went further by taking three childhood misfortune indicators (CSC, ACE, and ACHE) into account when studying frailty at older age.

With respect to differences in the associations with the odds of being (pre-)frail across the different welfare regimes, it seemed that the Bismarckian welfare regime is the least able to deal with cumulative disadvantage. Originally, this is a regime where social reproduction is high in terms of inequality in educational attainment (Allmendinger & Leibfried, 2003). Moreover, our results showed that childhood misfortune and educational attainment are all consistently associated with higher odds of (pre-)frailty in old age. The frailty trajectories were similarly impacted: initial inequalities have not been absorbed and they affect health in later life. Also Eastern Europe exhibited a large persistence of social inequalities along the life-course. Part of the explanation might be the disruption of social institutions and structures after the collapse of the Soviet regime, which resulted in an increase of mortality and a privatization of many sectors, including health. Therefore, participants in SHARE are survivors and it seems that their social distribution of health in old age depends on current conditions that are unable to overcome inequalities in early life (Cornia, 2016; Meslé, 2004). The most egalitarian welfare regimes in relation to frailty appear to be the Scandinavian, social equality being the objective of this regime since its onset (Eikemo et al., 2008) and the Southern European, despite the frequent criticisms against this regime. Another study on self-rated health also looked at these associations and their differences in these associations across welfare regimes (Sieber et al., in preparation). Results are similar to our findings on frailty: the more disadvantaged respondents in terms of childhood misfortune and adult-life SEC also experienced poorer self-rated health. In addition, the study further supported our finding of similar patterns across welfare regimes for associations of satisfaction with household income with health at older age and differing patterns for CSC, education, and occupation.

### Strengths and Limitations

A major strength of this study is the use of a longitudinal survey with 12-year follow-up with a large sample size. Additionally, the comprehensive multinational data on both childhood and adult-life indicators allowed us to study trajectories of frailty across different welfare regimes. This enabled us to do a comparative analysis testing three aspects of the CDA framework; growing differences in frailty trajectories over aging, interactive dynamics across the life-course, and the interactions of the micro and macro level from age 50 onwards (Dannefer, 2018).

This study has some limitations. First, data used for the childhood misfortune and adult-life SEC indicators was self-reported and measured retrospectively. This may be subject to recall bias and social desirability. However, studies on recall measures of adverse experiences and SEC in older adults have showed adequate validity (Barboza Solís et al., 2015; Lacey, Belcher, & Croft, 2012). Second, selection bias may have occurred as longitudinal data were used and participants have dropped out or died during follow-up. We adjusted our models for attrition to deal with this and we conducted a sensitivity analysis excluding participants who dropped out or died. Third, our measure of ACE is an overall score of adversity. Even though combining different indicators has been done in previous studies (Cheval et al., 2019), this does not enable us to disentangle the specific effect of each indicator, nor to explore a possible threshold of adversity. Fourth, other variables could be important in these analyses, such as the late-life adverse events, genetic predispositions, and other psychosocial factors. Unfortunately, data on this was not available. Finally, the definition and operationalization of frailty and CDA differs between studies, which may make comparisons between studies more difficult. The operationalization of frailty used in this study is similar to previous studies using SHARE data and seems to be the optimal choice.

## Conclusion

The present study is the first to analyze associations of childhood misfortune and adult-life SEC with frailty at older age. In addition, it is the first time that the influence of welfare regimes on these associations is studied. In conclusion, our findings suggest that childhood misfortune and adult-life SEC influence (pre-)frailty at older age. It also demonstrates that the effect of CSC, but not ACE and ACHE, is mediated by adult-life SEC, suggesting that adult-life SEC capture the cumulative disadvantage produced by CSC. Narrowing differences over aging were found for the different categories of ACE, which were driven by the Scandinavian welfare regime, and of adverse childhood health experiences, driven by the Eastern European welfare regime. For main occupational position an increased difference was found, which was driven by the Bismarckian welfare regime. In terms of conceptual conclusions for the CDA theory, apart from occupational position, after the age of 50 differences in frailty trajectories by the various childhood misfortune and adult-life SEC variables were narrowing, which does not support the CDA theory. These results show the importance of childhood as well as adult-life SEC on health in later life. Moreover, we demonstrate that policies of welfare regimes do not necessarily compensate for this.

## Funding

This work was supported by the European Union’s Horizon 2020 Research and Innovation Programme under the Marie Sklodowska-Curie Grant (agreement number 676060 [LONGPOP] to B. W. A. van der Linden); and the Swiss National Centre of Competence in Research “LIVES – Overcoming vulnerability: Life course perspectives”, which is financed by the Swiss National Science Foundation (grant number: 51NF40-160590). B. Cheval is supported by an Ambizione grant (PZ00P1_180040) from the Swiss National Science Foundation (SNSF). This article uses data from SHARE Waves 1, 2, 3 (SHARELIFE), 4, 5 and 6 (DOIs: 10.6103/SHARE.w1.600, 10.6103/SHARE.w2.600, 10.6103/SHARE.w3.600, 10.6103/SHARE.w4.600, 10.6103/SHARE.w5.600, 10.6103/SHARE.w6.600). The SHARE data collection was primarily funded by the European Commission through FP5 (QLK6-CT-2001-00360), FP6 (SHARE-I3: RII-CT-2006-062193, COMPARE: CIT5-CT-2005-028857, SHARELIFE: CIT4-CT-2006-028812), and FP7 (SHARE-PREP: N°211909, SHARE-LEAP: N°227822, SHARE M4: N°261982). The authors gratefully acknowledge additional funding from the German Ministry of Education and Research, the Max Planck Society for the Advancement of Science, the US National Institute on Aging (U01_AG09740-13S2, P01_AG005842, P01_AG08291, P30_AG12815, R21_AG025169, Y1-AG-4553-01, IAG_BSR06-11, OGHA_04-064, HHSN271201300071C) and various national funding sources (www.share-project.org).

## Author Contributions

B.W.A. van der Linden, S. Sieber, B. Cheval, and S. Cullati designed the analyses. B.W.A. van der Linden, B. Cheval, D. Orsholits, and S. Sieber were involved in analyzing the data. B.W.A. van der Linden drafted the manuscript and all authors helped to write and revise the manuscript.

## Conflict of Interest

None reported.

## Supplementary Material

gbz140_suppl_Supplementary_Material
