# Supplementary material for: Life-Course Circumstances and Frailty in Old Age Within Different European Welfare Regimes: A Longitudinal Study With SHARE
Source: J Gerontol B Psychol Sci Soc Sci. 2019 Oct 30;75(6):1326–35. doi: 10.1093/geronb/gbz140 (PMC7265805; doi:10.1093/geronb/gbz140)
Supplement: gbz140_suppl_Supplementary_Material [file gbz140_suppl_supplementary_material.docx]

**Life-course circumstances and frailty in old age within different European welfare regimes: a longitudinal study with SHARE**

Supplementary Material

Content

- **Table S1.** Participant characteristics for total and stratified sample by welfare regime
- **Table S2.** Associations of childhood misfortune and adult-life socioeconomic circumstances with level and trajectories of frailty at old age stratified by Scandinavian and Bismarckian welfare regime
- **Table S3.** Associations of childhood misfortune and adult-life socioeconomic circumstances with level and trajectories of frailty at old age stratified by Southern and Eastern European welfare regime
- **Table S4.** Fully adjusted associations (health- and lifestyle variables) of childhood misfortune and adult-life socioeconomic circumstances with level and trajectories of frailty at old age for total and stratified sample by welfare regime.

**Table S1. Participant characteristics for total and stratified sample by welfare regime**

|  | Total |  | Scandinavian | | Bismarckian | | Southern European | | Eastern European | |
| --- | --- | --- | --- | --- | --- | --- | --- | --- | --- | --- |
|  | Non-frail | (pre-)frail | Non-frail | (pre-)frail | Non-frail | (pre-)frail | Non-frail | (pre-)frail | Non-frail | (pre-)frail |
|  | N (%) | N (%) | N (%) | N (%) | N (%) | N (%) | N (%) | N (%) | N (%) | N (%) |
| Total | 11731 (50.2) | 11627 (49.8) | 2019 (56.2) | 1571 (43.8) | 5559 (55.3) | 4495 (44.7) | 2782 (57.7) | 3795 (42.3) | 1371 (43.7) | 1766 (56.3) |
| Age, mean (SD) | 60.6 (7.8) | 64.6 (9.8) | 61.2 (8.2) | 64.0 (9.9) | 60.9 (8.0) | 64.6 (10.0) | 59.8 (7.5) | 64.8 (9.5) | 60.0 (7.2) | 65.0 (9.7) |
| Sex |  |  |  |  |  |  |  |  |  |  |
| Female | 5954 (45.8) | 7038 (54.2) | 1030 (52.2) | 942 (47.8) | 2825 (50.8) | 2740 (49.2) | 1397 (38.2) | 2260 (61.8) | 702 (39.0) | 1096 (61.0) |
| Male | 5777 (55.7) | 4589 (44.3) | 989 (61.1) | 629 (38.9) | 2734 (60.9) | 1755 (39.1) | 1385 (47.4) | 1535 (52.6) | 669 (50.0) | 670 (50.0) |
| Birth cohort |  |  |  |  |  |  |  |  |  |  |
| After 1945 | 5941 (57.8) | 4330 (42.2) | 927 (60.7) | 601 (39.9) | 2672 (61.5) | 1672 (38.5) | 1506 (53.0) | 1338 (47.0) | 836 (53.8) | 719 (46.2) |
| Between 1939 and 1945 | 2983 (54.6) | 2479 (45.4) | 542 (60.0) | 361 (40.0) | 1430 (60.8) | 922 (39.2) | 679 (45.4) | 815 (54.6) | 332 (46.6) | 381 (53.4) |
| Between 1929 and 1938 | 2312 (42.4) | 3142 (57.6) | 421 (53.5) | 366 (46.5) | 1185 (50.4) | 1168 (49.6) | 527 (31.6) | 1141 (68.4) | 179 (27.7) | 467 (72.3) |
| Between 1919 and 1928 | 495 (22.8) | 1676 (77.2) | 129 (34.7) | 243 (65.3) | 272 (27.1) | 733 (72.9) | 70 (12.3) | 501 (87.7) | 24 (10.8) | 199 (89.2) |
| Attrition |  |  |  |  |  |  |  |  |  |  |
| No dropout | 8846 (52.3) | 8074 (47.7) | 1638 (58.4) | 1168 (41.6) | 4042 (57.6) | 2979 (42.4) | 2167 (43.8) | 2778 (56.2) | 999 (46.5) | 1149 (53.5) |
| Dropped | 2318 (52.2) | 2125 (47.8) | 263 (59.5) | 179 (40.5) | 1312 (53.8) | 1126 (46.2) | 479 (48.4) | 510 (51.6) | 264 (46.0) | 310 (54.0) |
| Deceased | 567 (28.4) | 1428 (71.6) | 118 (34.5) | 224 (65.5) | 205 (34.5) | 390 (65.5) | 136 (21.2) | 507 (78.8) | 108 (26.0) | 307 (74.0) |
| ACE |  |  |  |  |  |  |  |  |  |  |
| None | 9465 (51.5) | 8914 (48.5) | 1631 (57.7) | 1195 (42.3) | 4409 (56.6) | 3385 (43.4) | 2327 (44.2) | 2933 (55.8) | 1098 (43.9) | 1401 (56.1) |
| At least one | 2266 (45.5) | 2713 (54.5) | 388 (50.8) | 376 (49.2) | 1150 (50.9) | 1110 (49.1) | 455 (34.5) | 862 (65.5) | 273 (42.8) | 365 (57.2) |
| ACHE |  |  |  |  |  |  |  |  |  |  |
| None | 8723 (50.1) | 8698 (49.9) | 1405 (56.4) | 1086 (43.6) | 3967 (55.8) | 3146 (44.2) | 2329 (42.8) | 3113 (57.2) | 1022 (43.0) | 1353 (57.0) |
| At least one | 3008 (50.7) | 2929 (62.8) | 614 (55.9) | 485 (44.1) | 1592 (54.1) | 1349 (45.9) | 453 (39.9) | 682 (60.1) | 349 (45.8) | 413 (54.2) |
| CSC |  |  |  |  |  |  |  |  |  |  |
| Most disadvantaged | 1598 (37.2) | 2697 (62.8) | 87 (41) | 125 (59) | 480 (47.0) | 542 (53.0) | 742 (34.8) | 1388 (65.2) | 289 (31.0) | 642 (69.0) |
| Disadvantaged | 2810 (48.0) | 3041 (52.0) | 268 (49.8) | 270 (50.2) | 1225 (52.8) | 1094 (47.2) | 1006 (44.5) | 1256 (55.5) | 311 (42.5) | 421 (57.5) |
| Middle | 4097 (54.3) | 3454 (45.7) | 774 (56.0) | 609 (44.0) | 2041 (57.9) | 1486 (42.1) | 732 (47.2) | 820 (52.8) | 550 (50.5) | 539 (49.5) |
| Advantaged | 2448 (56.5) | 1886 (43.5) | 648 (60.3) | 426 (39.7) | 1350 (56.8) | 1027 (43.2) | 254 (47.3) | 283 (52.7) | 196 (56.6) | 150 (43.3) |
| Most advantaged | 778 (58.6) | 549 (41.4) | 242 (63.2) | 141 (36.8) | 463 (57.2) | 346 (42.8) | 48 (50.0) | 48 (50.0) | 25 (64.1) | 14 (35.9) |
| Education |  |  |  |  |  |  |  |  |  |  |
| Primary | 2560 (36.9) | 4380 (63.1) | 351 (49.4) | 360 (50.6) | 747 (40.8) | 1083 (59.2) | 1200 (34.4) | 2284 (65.6) | 262 (28.6) | 653 (71.4) |
| Secondary | 6396 (53.9) | 5462 (46.1) | 925 (55.4) | 746 (44.6) | 3335 (57.2) | 2497 (42.8) | 1194 (49.5) | 1217 (50.5) | 942 (48.5) | 1002 (51.5) |
| Tertiary | 2775 (60.9) | 1785 (39.1) | 743 (61.5) | 465 (38.5) | 1477 (61.7) | 915 (38.3) | 388 (56.9) | 294 (43.1) | 167 (60.1) | 111 (39.9) |
| Main occupational position |  |  |  |  |  |  |  |  |  |  |
| High skill | 3207 (60.0) | 2138 (40.0) | 808 (62.5) | 484 (37.5) | 1634 (61.9) | 1004 (38.1) | 409 (53.0) | 362 (47.0) | 356 (55.3) | 288 (44.7) |
| Low skill | 7876 (48.9) | 8242 (51.1) | 1201 (52.8) | 1074 (47.2) | 3788 (54.3) | 3184 (45.7) | 1895 (42.3) | 2588 (57.7) | 992 (41.5) | 1396 (58.5) |
| Never worked | 648 (34.2) | 1247 (65.8) | 10 (43.5) | 13 (56.5) | 137 (30.9) | 307 (69.1) | 478 (36.1) | 845 (63.9) | 23 (21.9) | 82 (78.1) |
| Satisfaction with household income |  |  |  |  |  |  |  |  |  |  |
| Easily | 5223 (59.4) | 3567 (40.6) | 1470 (59.8) | 989 (40.2) | 3034 (61.7) | 1881 (38.3) | 494 (49.5) | 503 (50.5) | 225 (53.7) | 194 (46.3) |
| Fairly easily | 3650 (51.1) | 3492 (48.9) | 431 (51.5) | 406 (48.5) | 1791 (53.1) | 1585 (46.9) | 892 (47.3) | 992 (52.7) | 536 (51.3) | 509 (48.7) |
| With some difficulty | 2093 (41.7) | 2924 (58.3) | 99 (41.6) | 139 (58.4) | 576 (44.2) | 727 (55.8) | 940 (41.3) | 1335 (58.7) | 478 (39.8) | 723 (60.2) |
| With great difficulty | 765 (31.8) | 1644 (68.2) | 19 (33.9) | 37 (66.1) | 158 (34.3) | 302 (65.7) | 456 (32.1) | 965 (67.9) | 132 (28.0) | 340 (72.0) |
| Partnership status |  |  |  |  |  |  |  |  |  |  |
| Alone | 2365 (41.3) | 3358 (58.7) | 414 (47.4) | 459 (52.6) | 1182 (44.5) | 1474 (55.5) | 449 (34.0) | 872 (66.0) | 320 (36.7) | 553 (63.3) |
| In couple | 9366 (53.1) | 8269 (46.9) | 1605 (59.1) | 1112 (40.9) | 4377 (59.2) | 3021 (40.8) | 2333 (44.4) | 2923 (55.6) | 1051 (46.4) | 1213 (53.6) |
| Delayed recall (SD) | 4.0 (1.9) | 4.0 (1.9) | 3.9 (1.8) | 3.9 (1.8) | 4.2 (1.9) | 4.1 (1.9) | 4.0 (1.9) | 4.1 (1.9) | 3.8 (1.8) | 3.7 (1.7) |
| Verbal fluency (SD) | 21.7 (6.9) | 21.7 (6.9) | 21.4 (6.9) | 21.2 (6.7) | 21.9 (7.1) | 22.0 (7.0) | 21.1 (6.6) | 21.3 (6.8) | 22.1 (6.9) | 21.9 (7.1) |
| Ever smoked (SD) | 0.5 (0.5) | 0.5 (0.5) | 0.6 (0.5) | 0.6 (0.5) | 0.5 (0.5) | 0.5 (0.5) | 0.5 (0.5) | 0.4 (0.5) | 0.5 (0.5) | 0.4 (0.5) |
| Number of chronic conditions (SD) | 1.1 (1.1) | 2.0 (1.6) | 1.1 (1.2) | 1.9 (1.5) | 1.1 (1.1) | 1.8 (1.5) | 1.1 (1.1) | 2.0 (1.6) | 1.3 (1.2) | 2.4 (1.7) |
| Difficulties ADL (SD) | 0.3 (0.7) | 0.2 (0.7) | 0.2 (0.6) | 0.2 (0.7) | 0.3 (0.7) | 0.2 (0.7) | 0.3 (0.8) | 0.2 (0.7) | 0.2 (0.6) | 0.2 (0.7) |
| Difficulties IADL (SD) | 0.4 (0.9) | 0.4 (0.9) | 0.4 (0.9) | 0.4 (1.0) | 0.4 (0.9) | 0.4 (1.0) | 0.4 (0.8) | 0.4 (0.9) | 0.4 (0.9) | 0.4 (0.8) |

*Note.* ACE, adverse childhood experiences; ACHE, adverse childhood health experiences; ADL, activities of daily living; CI, confidence interval; CSC, childhood socioeconomic conditions; IADL, instrumental activities of daily living; SD, standard deviation; WR, welfare regime.

**Table S2. Associations of childhood misfortune and adult-life socioeconomic circumstances with level and trajectories of frailty at old age stratified by Scandinavian and Bismarckian welfare regime**

| \|  \|  \| \| --- \| --- \| \|  \| **Scandinavian**  M1b \| \| M2b \|  \| **Bismarckian**  M1b \| M2b \| \|  \| OR (95% CI) \| \| OR (95% CI) \|  \| OR (95% CI) \| OR (95% CI) \| \| Age (10-y period) \| 2.14 (1.53-2.99)*** \| \| 2.08 (1.42-3.05)*** \|  \| 2.72 (2.34-3.18)*** \| 2.64 (2.17-3.20)*** \| \| At least one ACE^a^ \| 2.14 (1.56-2.92)*** \| \| 2.07 (1.51-2.82)*** \|  \| 1.55 (1.27-1.88)*** \| 1.39 (1.15-1.69)*** \| \| At least one ACHE^b^ \| 1.12 (0.84-1.48) \| \| 1.13 (0.85-1.49) \|  \| 1.53 (1.29-1.81)*** \| 1.49 (1.26-1.76)*** \| \| CSC^c^ \|  \| \|  \|  \|  \|  \| \| Disadvantaged \| 1.09 (0.45-2.65) \| \| 1.05 (0.43-2.57) \|  \| 1.05 (0.75-1.49) \| 1.16 (0.82-1.63) \| \| Middle \| 1.11 (0.48-2.53) \| \| 1.11 (0.48-2.59) \|  \| 0.92 (0.66-1.28) \| 1.19 (0.85-1.67) \| \| Advantaged \| 0.93 (0.41-2.14) \| \| 1.01 (0.43-2.37) \|  \| 1.17 (0.83-1.64) \| 1.63 (1.15-2.30)** \| \| Most advantaged \| 0.52 (0.21-1.26) \| \| 0.58 (0.23-1.45) \|  \| 0.97 (0.65-1.44) \| 1.41 (0.93-2.14) \| \| Education^d^ \|  \| \|  \|  \|  \|  \| \| Secondary \|  \| \| 1.10 (0.81-1.51) \|  \|  \| 1.16 (0.95-1.42) \| \| Primary \|  \| \| 0.65 (0.39-1.09) \|  \|  \| 1.81 (1.34-2.44)*** \| \| Main Occupational Position^e^ \|  \| \|  \|  \|  \|  \| \| Low skill \|  \| \| 1.65 (1.21-2.23)** \|  \|  \| 0.94 (0.78-1.15) \| \| Never worked \|  \| \| 1.00 (0.14-6.85) \|  \|  \| 1.53 (0.90-2.61) \| \| Satisfaction with household income^f^ \|  \| \|  \|  \|  \|  \| \| Fairly easily \|  \| \| 1.39 (1.01-1.91)* \|  \|  \| 1.57 (1.32-1.88)*** \| \| With some difficulty \|  \| \| 2.56 (1.44-4.55)** \|  \|  \| 2.94 (2.31-3.75)*** \| \| With great difficulty \|  \| \| 3.82 (1.23-11.86)* \|  \|  \| 7.35 (5.05-10.71)*** \| \| **Interactions** \|  \| \|  \|  \|  \|  \| \| Age x at least one ACE^a^ \| 0.75 (0.64-0.88)** \| \| 0.75 (0.64-0.89)** \|  \| 0.92 (0.83-1.02) \| 0.96 (0.87-1.06) \| \| Age x at least one ACHE^b^ \| 1.04 (0.89-1.20) \| \| 1.03 (0.89-1.19) \|  \| 0.91 (0.82-1.00)* \| 0.91 (0.83-0.99)* \| \| Age x CSC^c^ \|  \| \|  \|  \|  \|  \| \| Age x Disadvantaged \| 0.94 (0.66-1.35) \| \| 0.97 (0.67-1.39) \|  \| 0.95 (0.81-1.11) \| 0.96 (0.82-1.13) \| \| Age x Middle \| 0.81 (0.58-1.13) \| \| 0.85 (0.60-1.21) \|  \| 0.92 (0.79-1.08) \| 0.93 (0.80-1.09) \| \| Age x Advantaged \| 0.83 (0.59-1.17) \| \| 0.87 (0.61-1.25) \|  \| 0.81 (0.69-0.95)* \| 0.83 (0.70-0.98)* \| \| Age x Most advantaged \| 1.01 (0.69-1.49) \| \| 1.10 (0.73-1.65) \|  \| 0.87 (0.72-1.06) \| 0.91 (0.74-1.12) \| \| Age x Education^d^ \|  \| \|  \|  \|  \|  \| \| Age x Secondary \|  \| \| 1.03 (0.87-1.22) \|  \|  \| 0.95 (0.84-1.06) \| \| Age x Primary \|  \| \| 1.26 (0.99-1.60) \|  \|  \| 0.92 (0.79-1.07) \| \| Age x Main occupational position^e^ \|  \| \|  \|  \|  \|  \| \| Age x Low skill \|  \| \| 0.88 (0.75-1.02) \|  \|  \| 1.12 (1.00-1.25)* \| \| Age x Never worked \|  \| \| 1.24 (0.56-2.76) \|  \|  \| 1.05 (0.83-1.33) \| \| Age x Satisfaction with household income^f^ \|  \| \|  \|  \|  \|  \| \| Age x Fairly easily \|  \| \| 1.05 (0.90-1.24) \|  \|  \| 1.00 (0.91-1.10) \| \| Age x With some difficulty \|  \| \| 0.93 (0.71-1.23) \|  \|  \| 0.91 (0.79-1.04) \| \| Age x With great difficulty \|  \| \| 1.08 (0.58-2.00) \|  \|  \| 0.72 (0.58-0.91)** \| |  |  |
| --- | --- | --- | --- | --- | --- | --- | --- | --- | --- | --- | --- | --- | --- | --- | --- | --- | --- | --- | --- | --- | --- | --- | --- | --- | --- | --- | --- | --- | --- | --- | --- | --- | --- | --- | --- | --- | --- | --- | --- | --- | --- | --- | --- | --- | --- | --- | --- | --- | --- | --- | --- | --- | --- | --- | --- | --- | --- | --- | --- | --- | --- | --- | --- | --- | --- | --- | --- | --- | --- | --- | --- | --- | --- | --- | --- | --- | --- | --- | --- | --- | --- | --- | --- | --- | --- | --- | --- | --- | --- | --- | --- | --- | --- | --- | --- | --- | --- | --- | --- | --- | --- | --- | --- | --- | --- | --- | --- | --- | --- | --- | --- | --- | --- | --- | --- | --- | --- | --- | --- | --- | --- | --- | --- | --- | --- | --- | --- | --- | --- | --- | --- | --- | --- | --- | --- | --- | --- | --- | --- | --- | --- | --- | --- | --- | --- | --- | --- | --- | --- | --- | --- | --- | --- | --- | --- | --- | --- | --- | --- | --- | --- | --- | --- | --- | --- | --- | --- | --- | --- | --- | --- | --- | --- | --- | --- | --- | --- | --- | --- | --- | --- | --- | --- | --- | --- | --- | --- | --- | --- | --- | --- | --- | --- | --- | --- | --- | --- | --- | --- | --- | --- | --- | --- | --- | --- | --- | --- | --- | --- | --- | --- | --- | --- | --- | --- | --- | --- | --- | --- | --- | --- | --- | --- | --- | --- | --- | --- | --- | --- | --- | --- | --- | --- | --- | --- | --- | --- | --- | --- | --- | --- | --- | --- | --- | --- | --- | --- | --- | --- | --- | --- | --- | --- | --- | --- | --- | --- | --- | --- | --- | --- | --- | --- | --- | --- | --- | --- | --- | --- | --- |
| *Note.* ACE, adverse childhood experiences; ACHE, adverse childhood health experiences; CI, confidence interval; CSC, childhood socioeconomic conditions; OR, odds ratio. All models are adjusted for sex, birth cohort and attrition. Age was centered at 50 y and divided by 10 so that the coefficients yielded the effects for a 10-year period. N Scandinavian = 3590, N Bismarckian = 10054.  ^a^Adverse childhood experiences, reference category none  ^b^Adverse childhood health experiences, reference category none  ^c^Childhood socioeconomic conditions, reference category most disadvantaged  ^d^Education, reference category tertiary  ^e^Main occupational position, reference category high skill  ^f^Satisfaction with household income, reference category easily  ***p<.001, **p<.01, *p<.05 |  |  |
|  |  |  |
|  |  |  |
|  |  |  |

**Table S3.** **Associations of childhood misfortune and adult-life socioeconomic circumstances with level and trajectories of frailty at old age stratified by Southern and Eastern European welfare regime**

|  |  |  |  |  |  |  |
| --- | --- | --- | --- | --- | --- | --- |
|  | **Southern European**  M1b | | M2b |  | **Eastern European**  M1b | M2b |
|  | OR (95% CI) | | OR (95% CI) |  | OR (95% CI) | OR (95% CI) |
| Age (10-y period) | 2.42 (2.11-2.78)*** | | 2.11 (1.58-2.82)*** |  | 2.20 (1.78-2.73)*** | 2.35 (1.60-3.44)** |
| At least one ACE^a^ | 1.02 (0.75-1.37) | | 0.94 (0.70-1.26) |  | 1.02 (0.71-1.46) | 1.00 (0.70-1.42) |
| At least one ACHE^b^ | 1.21 (0.93-1.37) | | 1.24 (0.95-1.62) |  | 1.89 (1.39-2.59)*** | 1.71 (1.26-2.33)** |
| CSC^c^ |  | |  |  |  |  |
| Disadvantaged | 0.95 (0.72-1.26) | | 1.03 (0.78-1.37) |  | 0.80 (0.53-1.21) | 0.90 (0.60-1.36) |
| Middle | 0.86 (0.64-1.15) | | 1.05 (0.78-1.43) |  | 0.58 (0.40-0.84)** | 0.73 (0.50-1.07) |
| Advantaged | 0.76 (0.51-1.14) | | 1.00 (0.65-1.52) |  | 0.57 (0.35-0.92)* | 0.84 (0.51-1.38) |
| Most advantaged | 0.44 (0.19-1.07) | | 0.71 (0.29-1.72) |  | 0.40 (0.13-1.26) | 0.72 (0.23-2.27) |
| Education^d^ |  | |  |  |  |  |
| Secondary |  | | 1.35 (0.95-1.92) |  |  | 1.12 (0.67-1.87) |
| Primary |  | | 1.39 (0.94-2.05) |  |  | 1.07 (0.58-1.97) |
| Main Occupational Position^e^ |  | |  |  |  |  |
| Low skill |  | | 0.91 (0.64-1.28) |  |  | 1.32 (0.90-1.93) |
| Never worked |  | | 0.71 (0.47-1.08) |  |  | 1.26 (0.43-3.71) |
| Satisfaction with household income^f^ |  | |  |  |  |  |
| Fairly easily |  | | 1.12 (0.81-1.54) |  |  | 0.98 (0.64-1.50) |
| With some difficulty |  | | 1.48 (1.08-2.03)* |  |  | 1.81 (1.18-2.78)** |
| With great difficulty |  | | 2.59 (1.81-3.72)*** |  |  | 3.71 (2.19-6.30)*** |
| **Interactions** |  | |  |  |  |  |
| Age x at least one ACE^a^ | 1.14 (0.98-1.32) | | 1.15 (0.99-1.34) |  | 1.00 (0.82-1.23) | 1.00 (0.82-1.23) |
| Age x at least one ACHE^b^ | 1.02 (0.87-1.19) | | 1.00 (0.86-1.17) |  | 0.77 (0.63-0.93)** | 0.80 (0.66-0.97)* |
| Age x CSC^c^ |  | |  |  |  |  |
| Age x Disadvantaged | 0.90 (0.78-1.05) | | 0.90 (0.78-1.05) |  | 0.82 (0.65-1.03) | 0.84 (0.67-1.07) |
| Age x Middle | 0.89 (0.76-1.05) | | 0.92 (0.78-1.09) |  | 0.80 (0.65-0.99)* | 0.84 (0.67-1.05) |
| Age x Advantaged | 1.02 (0.80-1.29) | | 1.12 (0.87-1.44) |  | 0.75 (0.56-1.00)* | 0.75 (0.56-1.01) |
| Age x Most advantaged | 1.07 (0.67-1.70) | | 1.19 (0.73-1.93) |  | 0.88 (0.45-1.71) | 0.84 (0.43-1.64) |
| Age x Education^d^ |  | |  |  |  |  |
| Age x Secondary |  | | 1.00 (0.79-1.25) |  |  | 1.01 (0.75-1.37) |
| Age x Primary |  | | 1.15 (0.90-1.46) |  |  | 1.16 (0.81-1.65) |
| Age x Main occupational position^e^ |  | |  |  |  |  |
| Age x Low skill |  | | 1.05 (0.86-1.29) |  |  | 0.94 (0.74-1.17) |
| Age x Never worked |  | | 1.20 (0.95-1.52) |  |  | 1.46 (0.76-2.80) |
| Age x Satisfaction with household income^f^ |  | |  |  |  |  |
| Age x Fairly easily |  | | 0.95 (0.80-1.14) |  |  | 1.00 (0.79-1.28) |
| Age x With some difficulty |  | | 1.05 (0.88-1.26) |  |  | 0.85 (0.61-1.18) |
| Age x With great difficulty |  | | 0.99 (0.81-1.21) |  |  | 0.87 (0.68-1.11) |

*Note.* ACE, adverse childhood experiences; ACHE, adverse childhood health experiences; CI, confidence interval; CSC, childhood socioeconomic conditions; OR, odds ratio. All models are adjusted for sex, birth cohort and attrition. Age was centered at 50 y and divided by 10 so that the coefficients yielded the effects for a 10-year period. N Southern European = 6577, N Eastern European = 3137.

^a^Adverse childhood experiences, reference category none

^b^Adverse childhood health experiences, reference category none

^c^Childhood socioeconomic conditions, reference category most disadvantaged

^d^Education, reference category tertiary

^e^Main occupational position, reference category high skill

^f^Satisfaction with household income, reference category easily

***p<.001, **p<.01, *p<.05

**Table S4. Fully adjusted associations (health- and lifestyle variables) of childhood misfortune and adult-life socioeconomic circumstances with level and trajectories of frailty at old age for total and stratified sample by welfare regime**

|  | **Total Sample**  M3b |  | **Scandinavian**  M3b |  | **Bismarckian**  M3b |  | **Southern European**  M3b |  | **Eastern European**  M3b |
| --- | --- | --- | --- | --- | --- | --- | --- | --- | --- |
|  | OR (95% CI) |  | OR (95% CI) |  | OR (95% CI) |  | OR (95% CI) |  | OR (95% CI) |
| Age (10-y period) | 1.90 (1.68-2.14)*** |  | 1.75 (1.21-2.54)** |  | 2.12 (1.76-2.57)*** |  | 1.59 (1.20-2.11)** |  | 2.02 (1.40-2.93)*** |
| At least one ACE^a^ | 1.19 (1.05-1.35)** |  | 1.84 (1.36-2.47)*** |  | 1.33 (1.11-1.60)** |  | 0.86 (0.64-1.14) |  | 0.94 (0.67-1.33) |
| At least one ACHE^b^ | 1.26 (1.13-1.41)*** |  | 1.06 (0.81-1.38) |  | 1.35 (1.15-1.58)*** |  | 1.11 (0.86-1.44) |  | 1.49 (1.11-2.01)** |
| CSC^c^ |  |  |  |  |  |  |  |  |  |
| Disadvantaged | 0.99 (0.83-1.18) |  | 0.94 (0.40-2.22) |  | 1.15 (0.83-1.61) |  | 1.04 (0.80-1.37) |  | 0.87 (0.59-1.30) |
| Middle | 1.01 (0.85-1.21) |  | 1.05 (0.47-2.37) |  | 1.17 (0.85-1.62) |  | 1.08 (0.80-1.45) |  | 0.78 (0.54-1.13) |
| Advantaged | 1.17 (0.96-1.42) |  | 1.02 (0.45-2.32) |  | 1.61 (1.15-2.25)** |  | 0.98 (0.65-1.48) |  | 0.93 (0.58-1.50) |
| Most advantaged | 0.89 (0.69-1.16) |  | 0.55 (0.23-1.34) |  | 1.40 (0.94-2.09) |  | 0.65 (0.28-1.55) |  | 1.00 (0.33-3.00) |
| Education^d^ |  |  |  |  |  |  |  |  |  |
| Secondary | 1.11 (0.97-1.27) |  | 1.07 (0.79-1.44) |  | 1.12 (0.93-1.36) |  | 1.18 (0.84-1.66) |  | 1.24 (0.76-2.03) |
| Primary | 1.21 (1.01-1.46)* |  | 0.60 (0.36-0.98)* |  | 1.60 (1.19-2.13)* |  | 1.10 (0.76-1.61) |  | 1.17 (0.65-2.12) |
| Main Occupational Position^e^ |  |  |  |  |  |  |  |  |  |
| Low skill | 1.08 (0.95-1.23) |  | 1.43 (1.07-1.91)* |  | 0.98 (0.81-1.18) |  | 0.88 (0.63-1.23) |  | 1.31 (0.91-1.89) |
| Never worked | 1.14 (0.89-1.45) |  | 1.06 (0.17-6.63) |  | 1.50 (0.90-2.52) |  | 0.75 (0.50-1.13) |  | 1.57 (0.55-4.45) |
| Satisfaction with household income^f^ |  |  |  |  |  |  |  |  |  |
| Fairly easily | 1.35 (1.19-1.53)*** |  | 1.40 (1.03-1.89)* |  | 1.49 (1.26-1.77)*** |  | 1.14 (0.84-1.56) |  | 0.98 (0.65-1.49) |
| With some difficulty | 2.01 (1.74-2.32)*** |  | 2.09 (1.20-3.63)** |  | 2.44 (1.93-3.09)*** |  | 1.48 (1.09-2.00)* |  | 1.60 (1.06-2.42)* |
| With great difficulty | 3.45 (2.84-4.19)*** |  | 2.58 (0.87-7.67) |  | 4.99 (3.46-7.18)*** |  | 2.48 (1.75-3.51)*** |  | 2.57 (1.54-4.30)*** |
| **Interactions** |  |  |  |  |  |  |  |  |  |
| Age x at least one ACE^a^ | 0.97 (0.91-1.04) |  | 0.76 (0.65-0.89)** |  | 0.93 (0.85-1.03) |  | 1.17 (1.01-1.36)* |  | 1.02 (0.84-1.25) |
| Age x at least one ACHE^b^ | 0.93 (0.88-0.99)* |  | 1.02 (0.89-1.18) |  | 0.92 (0.84-1.01) |  | 1.03 (0.88-1.20) |  | 0.83 (0.69-1.00)* |
| Age x CSC^c^ |  |  |  |  |  |  |  |  |  |
| Age x Disadvantaged | 0.92 (0.85-1.01) |  | 1.01 (0.71-1.43) |  | 0.95 (0.81-1.11) |  | 0.91 (0.79-1.05) |  | 0.87 (0.69-1.09) |
| Age x Middle | 0.89 (0.81-0.97)** |  | 0.89 (0.64-1.25) |  | 0.94 (0.81-1.09) |  | 0.93 (0.79-1.09) |  | 0.84 (0.68-1.04) |
| Age x Advantaged | 0.84 (0.75-0.92)** |  | 0.89 (0.63-1.25) |  | 0.81 (0.69-0.96)* |  | 1.14 (0.89-1.46) |  | 0.74 (0.56-0.99)* |
| Age x Most advantaged | 0.97 (0.84-1.12) |  | 1.15 (0.78-1.71) |  | 0.91 (0.75-1.12) |  | 1.28 (0.80-2.06) |  | 0.87 (0.46-1.66) |
| Age x Education^d^ |  |  |  |  |  |  |  |  |  |
| Age x Secondary | 1.00 (0.92-1.08) |  | 0.81 (0.67-0.99)* |  | 0.98 (0.87-1.09) |  | 1.04 (0.83-1.30) |  | 0.97 (0.73-1.29) |
| Age x Primary | 1.08 (0.98-1.19) |  | 0.81 (0.64-1.02) |  | 0.97 (0.83-1.12) |  | 1.20 (0.95-1.52) |  | 1.11 (0.79-1.57) |
| Age x Main occupational position^e^ |  |  |  |  |  |  |  |  |  |
| Age x Low skill | 1.03 (0.96-1.11) |  | 0.90 (0.77-1.06) |  | 1.08 (0.97-1.20) |  | 1.08 (0.89-1.32) |  | 0.93 (0.74-1.16) |
| Age x Never worked | 1.14 (1.01-1.30)* |  | 1.22 (0.57-2.63) |  | 1.02 (0.81-1.28) |  | 1.20 (0.95-1.51) |  | 1.28 (0.68-2.41) |
| Age x Satisfaction with household income^f^ |  |  |  |  |  |  |  |  |  |
| Age x Fairly easily | 1.03 (0.96-1.10) |  | 1.03 (0.88-1.20) |  | 0.99 (0.90-1.08) |  | 0.96 (0.80-1.14) |  | 0.97 (0.77-1.22) |
| Age x With some difficulty | 0.96 (0.86-1.07) |  | 0.93 (0.71-1.21) |  | 0.88 (0.78-1.01) |  | 1.02 (0.86-1.21) |  | 0.86 (0.68-1.09) |
| Age x With great difficulty | 1.00 (0.93-1.08) |  | 1.08 (0.60-1.95) |  | 0.74 (0.60-0.92)** |  | 0.96 (0.79-1.17) |  | 0.90 (0.67-1.28) |

*Note.* ACE, adverse childhood experiences; ACHE, adverse childhood health experiences; CI, confidence interval; CSC, childhood socioeconomic conditions; OR, odds ratio. All models are adjusted for sex, birth cohort, attrition, living with a partner, delayed recall memory, verbal fluency, smoking, number of chronic conditions, difficulties with activities of daily living, and difficulties with instrumental activities of daily living. Age was centered at 50 y and divided by 10 so that the coefficients yielded the effects for a 10-year period. N total sample = 23358, N Scandinavian = 3590, N Bismarckian = 10054, N Southern European = 6577, N Eastern European = 3137.

^a^Adverse childhood experiences, reference category none

^b^Adverse childhood health experiences, reference category none

^c^Childhood socioeconomic conditions, reference category most disadvantaged

^d^Education, reference category tertiary

^e^Main occupational position, reference category high skill

^f^Satisfaction with household income, reference category easily

***p<.001, **p<.01, *p<.05
